# Codevelopment of Implementation Interventions to Support Parent-Led Care for Pain in Infants: Protocol for a Qualitative Descriptive Study

**DOI:** 10.2196/33770

**Published:** 2022-08-05

**Authors:** Britney Benoit, Christine Cassidy, Jacqueline van Wijlen, Quinn Correll, Marsha Campbell-Yeo, Sionnach Hendra, Ruth Martin-Misener, Jennifer MacDougall, Ashley Cameron

**Affiliations:** 1 Rankin School of Nursing St Francis Xavier University Antigonish, NS Canada; 2 School of Nursing Dalhousie University Halifax, NS Canada; 3 Nova Scotia Health Antigonish, NS Canada

**Keywords:** breastfeeding, chestfeeding, skin-to-skin contact, infant pain, implementation, qualitative

## Abstract

**Background:**

Untreated pain in infants is associated with adverse health outcomes. Despite strong evidence for accessible, effective, and low-cost parent-led pain-relieving interventions such as breastfeeding or chestfeeding and skin-to-skin contact, these interventions are not routinely used.

**Objective:**

The objective of this study is to support the implementation of parent-led pain interventions by identifying barriers to and facilitators of parent-led, evidence-informed pain care in infants during acute procedures. In addition, this study aims to develop theory-informed, contextually relevant implementation interventions for supporting the use of parent-led pain care for infants in hospital and community contexts.

**Methods:**

This study will consist of 2 phases that follow a systematic, theoretically informed approach guided by the Theoretical Domains Framework and Behavior Change Wheel. In phase 1, we will use a qualitative descriptive design to explore barriers and facilitators to using parent-led pain care in infants from the perspectives of hospital and community-based clinicians, clinical leaders, and families. In phase 2, we will use the Behavior Change Wheel to design tailored implementation interventions that have evidence for effectively addressing identified barriers in collaboration with an advisory committee of administrative, clinical, and family leaders.

**Results:**

Ethics approval for this study was obtained in December 2020. As of May 2022, a total of 15 participants have been enrolled in phase 1. The results from all phases will be reported in 2023.

**Conclusions:**

Following the completion of this study, we will have co-designed theoretically informed implementation interventions that can be pilot-tested and experimentally applied. The findings will be used to implement parent-led interventions that improve patient safety and health outcomes for diverse families.

**International Registered Report Identifier (IRRID):**

DERR1-10.2196/33770

## Introduction

### Gender-neutral Infant Feeding Language

We are conscious of perpetuating oppressive and harmful discourses that do not reflect all childbearing families cared for in our health system and communities [1-3]. Therefore, we used inclusive gender-neutral language whenever possible throughout this protocol. This includes the use of terms such as *breastfeeding* or *chestfeeding* to better reflect diverse lactation experiences [3].

### Incidence and Outcomes of Acute Pain Exposure in Infants

All infants experience pain as part of routine care, both in hospitals and in the community. For example, all infants undergo a routine intramuscular injection of vitamin K to prevent bleeding [4] and a heel lance procedure to collect blood for metabolic testing and routine serum bilirubin screening [5] in the first hours of life. Repeated heel lancing is required in infants diagnosed with common clinical concerns such as hyperbilirubinemia [5] or hypoglycemia [6]. Children in Canada additionally undergo multiple necessary intramuscular injections for immunization, with the majority occurring between 2 and 18 months of age [7]. Studies examining the effects of untreated pain in infants have linked this exposure to adverse cardiorespiratory, hormonal, and neurodevelopmental effects [8-18]. In preterm infants, pain is associated with increased stress and inflammatory hormone release, which impede growth and tissue repair [8,9]. The motor, cognitive, and behavioral effects of untreated pain in preterm infants include poor growth of the body and head [15], reduced visual perceptual abilities [16], poorer language outcomes [17], and greater internalizing behaviors (anxiety and depression symptoms) [10,18] throughout childhood. In full-term infants, structural and functional alterations in both the peripheral and central nervous systems have been linked to both short- and long-term alterations in pain processing, most notably, increased sensitivity to pain during later procedures [11-14,19].

### Parent-Led Pain Care in Infants: Breastfeeding or Chestfeeding and Parent-Infant Skin-to-Skin Contact

In light of these adverse consequences of infant pain, intensive scientific efforts have been undertaken to determine effective pain-reducing treatments. Although pharmacological agents such as opioids and topical anesthetics [20,21] have been studied, there is limited evidence for their safety and pain-reducing efficacy for the routine acute painful procedures that infants commonly undergo. In contrast, parent-led interventions are low-cost and have strong evidence of pain-reducing efficacy and safety [22-24]. In our most recent systematic reviews of breastfeeding [22] and parent-infant skin-to-skin contact [24] as interventions for procedural pain, we found that these interventions have the strongest evidence for reducing pain associated with acute tissue-breaking procedures.

### Barriers to Parent-Led Pain Management in Infants

#### Overview

Most of the evidence describing pain management practices in infants is in the neonatal intensive care unit (NICU) environment. Although both nurses and parents report positive perceptions regarding the pain-reducing effectiveness of breastfeeding and skin-to-skin contact for infants [25,26], uptake and sustained implementation of these interventions in clinical practice has been limited [25,27], with less than half of the infants receiving any form of pain-relieving treatment during tissue-breaking procedures [27]. Common reasons for not using these interventions include lack of knowledge, stress and anxiety, gatekeeping and parent exclusion, and challenges associated with the physical environment [25].

#### Lack of Knowledge

Lack of knowledge about pain management in infants has been identified as a barrier to evidence-informed pain care [28-30]. Parents reported feeling apprehensive about participating in pain relief methods as they were not informed of pain in infants and nonpharmacological pain management approaches, including skin-to-skin contact and breastfeeding [24]. Parents reported that resources such as educational pamphlets, videos, workshops, or active counseling as educative initiatives for parent-led pain management in infants would be useful to enhance their awareness of the importance and use of parent-led pain-reducing strategies [28,31,32]. Health care providers, including nurses and physicians, may lack the communication skills needed to effectively relay information about pain in infants to families under their care [33]. A study found that educational pamphlets were used but only as part of the patient’s discharge package [32]. Parents who lacked knowledge regarding parent-led pain management interventions in infants stated that to be appropriately educated, the health care team needed to improve on how and when information was given to them [25].

#### Stress and Anxiety

Stress and anxiety are also barriers to parental involvement in pain management in infants [28,29]. Parents who lack knowledge of pain relief interventions have been found to feel anxious and uncertain about their ability to provide pain relief [29]. Multiple studies have shown that parents in particular found it stressful to be present during painful procedures, either because of their own phobia and fear of needles or because it was emotionally very difficult to watch their infant in pain [28-30].

#### Gatekeeping and Parental Exclusion

The attitudes and behaviors of health care providers influence the abilities of parents to participate in parent-led pain management in infants [30]. Health care providers have reported feeling responsible for pain management, acting in a gatekeeper role by deciding *who* provides pain relief measures and *how* they are provided [28-30]. Health care providers may exclude parents from participating in painful procedures, because they underestimate the abilities of the parents or feel as though they are protecting the parents from fear or anxiety [28]. Some studies have shown that staff members excluded parents from being involved in pain relief during painful procedures, because their presence was seen as an additional stressor to the health care provider [29,31]. 

#### Physical Environment

Studies in this area have been conducted in NICU settings. The physical environment of the NICU acts as a barrier to parent-led pain management in infants [28,29]. Parents may struggle to find their role as caregivers in restrictive medicalized environments [28]. Technology and equipment, including incubators, act as barriers that limit the ability of parents to participate in pain relief measures [29]. The NICU has been described as lacking physical space for parents to be present [28] and has policies that prevent them from being present during reports or medical rounds, thus restricting access to their baby [29].

### Facilitators of Parent-Led Pain Management in Infants

Three facilitators have been identified to support parent-led pain management in infants in the literature, including motivation of parents to participate, the physical environment, and access to information.

#### Motivation to Participate

The main facilitator of parent-led pain management in infants identified is the motivation of parents to be active participants in pain relief strategies and their eagerness to be educated on the subject [28-30,34]. Parents of infants in the NICU found that seeing their infant in pain increased their desire to be involved in their care and pain reduction [29]. Health care providers who showed a positive attitude toward parental involvement in pain management in infants and who empowered parental education, influenced the motivation of parents to participate in strategies of parent-led pain management in infants [28,29,31]. Parents were more likely to be involved in pain management in infants when they wanted knowledge about pain in infants and felt responsible for the well-being of their infant [28].

#### Physical Environment

Although studies have identified the physical environment as a barrier to parent-led pain management in infants, some studies have shown that it is also a facilitator. Parents felt more comfortable participating in pain relief strategies when the physical environment was *family-friendly* [28]. Parents also stated that private rooms and kangaroo care chairs promoted participation [29]. 

#### Accessibility to Information and Clear Communication

Access to educational tools and information, as well as open communication between parents and staff about pain management in infants has been reported to promote parent participation [28,34]. Parents who had access to educational tools, such as pamphlets or videos, felt more prepared to participate in parent-led pain management [29]. A study suggested multiple ways of disseminating this information, including during birthing or parenting classes, in hospitals or physician’ offices, and in waiting rooms [34]. Parents were also more likely to participate in parent-led pain management, if the information used to educate them was obtained from a credible source [34]. Family-centered care approaches, in which health care providers partnered with parents on pain management in infants, promoted parental participation [31]. Health care providers who communicated appropriate timing and tasks for parent-led pain management enabled parents to be open to participating in pain relief [29].

### Rationale for This Study and Study Objectives

Overall, the literature highlights numerous barriers, facilitators, and opportunities to support parent-led pain care in infants, with a focus on NICU settings. However, limited research has been conducted that aims to better support the uptake of these best-practice interventions for infants cared for outside neonatal units, particularly in community settings. Most infants undergo routine painful procedures as part of healthy infant care delivered by postpartum clinical services, primary care providers, and community public health offices. Therefore, to promote positive outcomes, health care safety, and access to best-practice pain care, it is imperative that strategies that support the sustained implementation of parent-led pain care in infants be identified in diverse hospital and community care environments.

Furthermore, parent-led pain care may be hindered by ineffective implementation strategies in the local context. Successful implementation of evidence-informed practices relies on a comprehensive understanding of the barriers and facilitators to change and tailoring implementation interventions to the local context [35]. The use of theory can assist in identifying potential behavioral determinants that influence implementation. Subsequently, implementation interventions can be tailored to specific behavioral determinants and as a result, will likely bring about change [36]. To date, a theoretically informed approach to identifying behavioral determinants and developing tailored implementation interventions has not been described in the literature. To address this gap, the aims of this study are to (1) identify barriers to and facilitators of parent-led evidence-informed pain care in infants (ie, breastfeeding or chestfeeding and skin-to-skin contact) during routine acute procedures and (2) develop theory-informed, contextually relevant implementation interventions to support the use of evidence-informed pain care in infants in community- and hospital-care contexts.

## Methods

### Theoretical Framework

**Figure 1:**
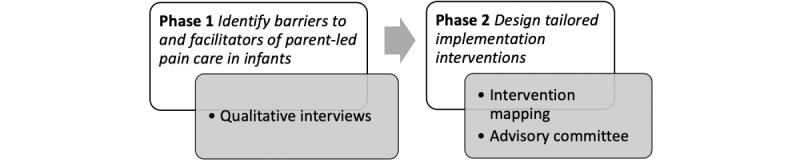
Study phases.

This study will consist of 2 phases (Figure 1) that follow a systematic, theoretically informed approach guided by the Theoretical Domains Framework (TDF) [36,37] and the Behavior Change Wheel (BCW) [38] to understand the barriers and facilitators and to design tailored implementation interventions.

The TDF is an integrated framework that provides a robust guide for implementation studies [36-39]. It has been previously used to identify barriers to and facilitators of evidence use in different health care contexts [36,40]. In addition, it has also been used to identify empirically tested implementation strategies to support evidence use [38]. The BCW is a systematic intervention design guide that pairs with the TDF to design tailored implementation interventions [38]. We use theory to guide this qualitative implementation study as it supports comprehensive identification of barriers to and facilitators of behavior change as well as development of complex and evidence-informed interventions to target barriers and enablers identified by participants [38]. A description of the phases of this study have been provided in the subsequent sections.

### Phase 1: Identify Barriers to and Facilitators of Parent-Led Pain Care in Infants

In phase 1, we will use a qualitative descriptive design [41], using one-on-one interviews to explore the barriers to and facilitators of using parent-led pain care during acute procedures.

#### Setting and Sample

Our setting includes regional hospitals and community-based contexts that provide perinatal and infant care in northeastern Nova Scotia, Canada. To obtain diverse perspectives, we will use stratified purposive sampling [36,42] of hospital and community-based health care providers (eg, acute care nurses, public health nurses, family practice nurses, acute and primary care physicians or nurse practitioners, midwives, obstetricians, and laboratory technicians), clinical leaders (eg, lactation consultants), administrators and policy makers (eg, program managers), and families who have used hospital or community perinatal services in northeastern Nova Scotia in the last 12 months. Face-to-face, semistructured, in-person interviews with each consenting participant will be conducted. We will aim to recruit approximately 20 to 30 [36,42,43] participants to obtain in-depth data related to the implementation of parent-led pain care. The large sample size was selected based on the heterogeneity of the target sample. To ensure that we have adequate representation and achieve data saturation across diverse health systems and experiences of parent participants, we will use qualitative sampling criteria [44] (which include evaluation of data variations, contraindications, and clarity) to determine if additional targeted recruitment of specific participant groups and exploration of specific experiences is needed to achieve depth and richness in the data. To recruit health system participants, a recruitment email containing study details and an invitation to participate in the study will be circulated through key research team partners and networks. For parent participants, electronic recruitment posters will be circulated via social media, and hard copy recruitment posters will be distributed through hospital and community antenatal care contexts across the province. We will strategically and purposively sample participants who identify as members of equity-seeking groups with diverse intersections of identity, represented across sex, gender, ethnicity, socioeconomic status, immigration or migration status, sexual orientation, ability status, and geography.

#### Data Collection

The TDF domains [36,37] were used to develop the semistructured interview questionnaires (Textboxes 1 and 2) and guide the analysis of participant interviews. The study interview guides were piloted with a parent partner and a health care provider partner to ensure the appropriateness and adequacy of the interview questions and the feasibility of completing the interview questions within a 60- to 90-minute time frame. Minor revisions to both interview guides were made based on partner feedback. One-on-one semistructured interviews are completed with each consenting participant.

**Study interview guide (family participants).**

**Knowledge**

- Have you or others you know used breast/chestfeeding and/or skin-to-skin contact to manage your babies’ pain? Tell me a little bit about that experience/what you know about using breast/chestfeeding or skin-to-skin contact to manage your baby’s pain.
- How do/did you find information about using breast/chestfeeding or skin-to-skin contact for managing your baby’s pain?

**Skill**

- What knowledge or supports do you use to breastfeed or provide skin-to-skin contact to your baby during pain? Is there additional knowledge or support that you need to breastfeed or provide skin-to-skin contact to your baby during pain?

**Intentions and goals**

- How important do you feel it is for your baby to be breast/cheastfed or be in held skin-to-skin contact during pain?

**Beliefs about consequences**

- Are there any benefits to using breast/chestfeeding or skin-to-skin contact to manage your baby’s pain? Are there any negatives to using breast/chestfeeding or skin-to-skin contact to manage your baby’s pain?

**Environmental context and resources**

- What factors influence you to use skin-to-skin contact or breast/chestfeeding to manage your baby’s pain? (Prompt(s): stressors, resources, barriers, or facilitators)

**Beliefs about capabilities**

- How confident do you feel in your ability to breastfeed or provide skin-to-skin contact to manage your baby’s pain? (Prompt: Is there anything that would make you more confident?)
- Are there challenges related to breast/chestfeeding or providing skin-to-skin contact for your baby when they are in pain? (Prompt(s): Is there anything that would make using breast/chestfeeding/skin-to-skin contact for your baby during pain easier?)

**Social influences**

- Do your family/friends influence your decision to use breast/chestfeeding or skin-to-skin contact to manage your baby’s pain? (Prompt(s): If yes, how would they influence your decision? To what extent do they influence your decision?)

**Emotion**

- Do emotions, both positive or negative, influence your decision to use skin-to-skin contact or breast/chestfeeding for your baby’s pain management? (Prompt(s): fear of consequences of using/not using skin-to-skin contact or breast/chestfeeding, anxiety, or stress).

**Conclusion**

- Are there any other key things related to using breast/chestfeeding or skin-to-skin contact to manage your baby’s pain that were not discussed today that you think are important to talk about?

**Study interview guide (health care provider and administrator participants).**

**Knowledge**

- Have you or others you know used breast/chestfeeding and/or skin-to-skin contact to manage infant pain? Tell me a little bit about that experience/what you know about using breast/chestfeeding or skin-to-skin contact to manage infant pain.
- How do/did you find information about using breast/chestfeeding or skin-to-skin contact to manage infant pain?

**Skill**

- What knowledge, resources, or skills do you use to support breast/chestfeeding and/or skin-to-skin contact to manage infant pain? Is there additional knowledge, resources, or skills that you need to support breast/chestfeeding and/or skin-to-skin contact to manage newborn pain?

**Intentions and goals**

- How important do you think it is for infants to have breast/chestfeeding or be held in skin-to-skin contact for pain management during procedures? If important, what actions have you taken toward using these strategies for pain management?

**Beliefs about consequences**

- Are there any benefits to using breast/chestfeeding or skin-to-skin contact to manage infant pain? Are there any negatives or harms to using breast/chestfeeding or skin-to-skin contact to manage infant pain?

**Environmental context and resources**

- What factors influence your decision or ability to use skin-to-skin contact or breast/chestfeeding for pain management in infants? (Prompt(s): stressors, resources, organizational culture, barriers, or facilitators).

**Beliefs about capabilities**

- How confident do you feel in your ability to support breast/chestfeeding or skin-to-skin contact to manage infant pain? (Prompt: Is there anything that would make you more confident?)
- Are there challenges related to supporting breast/chestfeeding or skin-to-skin contact for infants during painful procedures? (Prompt(s): Is there anything that would make supporting breast/chestfeeding/skin-to-skin contact for infants during pain easier?)

**Social/professional role identity**

- Do you feel like you have a responsibility to use pain management strategies for infants? Why or why not?
- Have you or others you know acted as a leader to support breast/chestfeeding and/or skin-to-skin contact for pain management in infants? (Prompt: If yes, what does that leadership look like in your organization and/or experience?)

**Social influences**

- How do your colleagues influence your decision to support breast/chestfeeding or skin-to-skin contact to manage pain in infants? (Prompt(s): To what extent do they influence your decision?)

**Reinforcement**

- Are there any incentives for you to support skin-to-skin contact or breast/chestfeeding for pain management in infants?

**Emotion**

- Do emotions influence your decision to support skin-to-skin contact or breast/chestfeeding for pain management in infants? (Prompt(s): fear of consequences of using/not using skin-to-skin contact or breast/chestfeeding, anxiety, stress, or burnout)

**Conclusion**

- Are there any other key things related to supporting breast/chestfeeding or skin-to-skin contact to manage infant pain that were not discussed today that you think are important to talk about?

#### Data Analysis

The transcriptions of audio-recorded interviews will undergo inductive-deductive qualitative content analysis [45-47] using NVivo (QSR International) qualitative data analysis software [48]. We will specifically use an intersectionality tool developed for use alongside the TDF [49] to support sex- and gender-based+ analysis. This tool includes intersectionality prompts for each of the 14 TDF domains to be used in participant interviews and data analysis to draw out information on the influences of social factors and structures of power on the implementation of parent-led pain care in infants [49]. First, 2 reviewers (BB and at least one other author) will deductively categorize [46,47] participant responses in the interview data into the 14 TDF domains [37]. Second, principles of inductive qualitative content analysis [46,47] will be used to generate categories and subcategories of salient barriers and facilitators related to the implementation of parent-led pain care within each of the relevant TDF domain categories [46]. To do this, participant responses will be read multiple times to identify the main points being addressed in relation to the TDF domains. Responses will be read line by line to generate codes, and these codes will be synthesized into higher level categories of barriers and enablers relevant to each of the TDF domains. Strategies to ensure trustworthiness in qualitative research [50,51] and implementation studies [52] will be used. Such approaches include clearly documenting and reporting the analysis process [45,52] and the culture, context, and selection and characteristics of the included participants [42,45,50,52]. Research participants will also be asked to provide feedback on the findings of the analysis (during the advisory committee meetings in phase 2) to ensure that they accurately represent experiences [45,50,51].

It is anticipated that the data from this diverse group of clinicians, clinical leaders, administrators, policy makers, and parents will highlight key behavioral determinants for interventions to support the use of parent-led pain care in infants which are in practice in hospitals and in the community.

### Phase 2: Develop Theory-Informed, Contextually Relevant Implementation Interventions

#### Overview

Phase 2 of this study will build on the findings of phase 1 to develop theoretically robust, empirically tested implementation interventions aimed at supporting the identified facilitators and overcoming the barriers to the use of parent-led pain care in infants. These interventions will be tested in subsequent studies. To do this, we will use the BCW [38], a systematic guide that pairs with the TDF to design tailored implementation interventions. We will implement a 2-step approach to the intervention design.

#### Phase 2(a): Mapping of Implementation Interventions

First, our research team will review the findings from phase 1 interviews alongside the BCW. The BCW provides 9 intervention functions (eg, education and environmental restructuring) that provide evidence for effectively changing behaviors in each TDF domain. We will map the relevant barriers and facilitators identified by participants within each of the TDF domains onto the intervention functions. Next, the BCW will be further used to map the intervention functions onto key “active ingredient” intervention components to create tailored interventions that target the identified barriers and facilitators in participant interviews.

#### Phase 2(b): Advisory Committee

Next, we will hold two 3-hour meetings with an advisory committee of several key administrative, clinical, and parent stakeholders who participated in phase 1 interviews to critically review the findings from phase 1 and the implementation interventions identified in phase 2(a). We will strategically invite committee members to ensure diverse and intersecting representation based on sex, gender, ethnicity, socioeconomic status, immigration or migration status, sexual orientation, ability status, and geography, ensuring a minimum of 2 parent stakeholders.

First, the research team will present the advisory committee the findings from phases 1 and 2(a) as a foundation for the refinement of implementation strategies. Second, a facilitator will lead the advisory committee to critically review the findings from phase 1 and the implementation interventions identified in phase 2(a) using the affordability, practicability, effectiveness and cost-effectiveness, acceptability, safety, and equity intervention criteria [38]. All discussion details will be documented by the study research assistant, consistent with the intervention development guidelines [52]. Strategies will be used to encourage authentic engagement and participation from all members of the advisory committee [53]. Such strategies will include using targeted questions for specific participant groups (ie, clinical stakeholders and parent stakeholders) to ensure feedback is obtained from all participants. In addition, small breakout groups will be used to facilitate targeted discussions, and participants will be encouraged to share verbal or written individual feedback or notes after the meeting has ended, if they are more comfortable doing so [53]. This discussion will help identify intervention feasibility and options for intervention delivery in different care environments and provide an opportunity to identify and refine details of optimal intervention implementation (eg, content, settings, recipients, providers, intensity, duration, and fidelity).

Following the completion of phase 2, we will have co-designed theoretically informed implementation interventions that have evidence for effectively supporting evidence implementation. These implementation interventions can subsequently be pilot-tested and experimentally applied in future studies to support the use of parent-led pain care for infants in both hospital and community contexts.

### Patient Engagement

Supporting the use of parent-led pain management strategies for infants has been identified as a clinical priority by parents in our previous work [26,54], and they will be engaged across the phases of this study. Following recommendations for patient and caregiver engagement from the Canadian Institutes of Health Research Strategy for Patient Oriented Research Patient Engagement Framework [55] and Health Quality Ontario [56], we have a dedicated parent partner as a member of our research team who is and will be engaged throughout the entire research process to ensure that parent or family perspectives and voice are well represented. Parents will be interviewed to identify their perspectives on barriers to and facilitators of using parent-led pain care in infants and will be regularly consulted to provide feedback on interpretation of the interview data throughout the data analysis. In addition, we will engage parents as members of our advisory committee. Parents will be supported to actively contribute to discussion regarding the adaptation and application of interventions to support the use of parent-led pain care in infants. Parent participants will be compensated for their contribution to study interviews and advisory committee work based on a parent partner compensation policy detailed by Solutions for Kids in Pain, a Canadian knowledge mobilization network [57]. Across all phases of this work, we will specifically engage families with diverse perspectives to provide a rich understanding of the complex ways in which equity, diversity, and inclusion influence the use of parent-led pain care in infants. It is anticipated that by engaging parents in this study, we will build relationships with parents who can be engaged as partners in subsequent research projects.

### Research Team

Our collaborative research team consists of clinicians, scientists, and administrators supporting service delivery to infants cared for in the community and acute care settings in northeastern Nova Scotia. Our team has expertise in pain assessment and management in infants, breast(chest)feeding promotion and support, parental interventions for pain in infants, maternal-child health, knowledge translation, implementation science, and acute and primary care. Our team includes a parent partner, and we have buy-in from key clinical and administrative collaborators in participating public health and hospital units.

### Ethics Approval

Ethics approval for this study was obtained from the Nova Scotia Health Research Ethics Board in December 2020 (#1026212).

## Results

As of May 2022, we have enrolled 15 participants in phase 1 of this study.

## Discussion

### Overview

Supporting the use of parent-led pain care in infants is essential for positive parent and infant health outcomes. This study will follow a systematic and theoretically informed approach to comprehensively map the barriers to and facilitators of parent-led pain care in infants in diverse hospital- and community-based practice contexts. These identified barriers and facilitators will inform the development of co-designed, theoretically informed implementation interventions tailored to a variety of clinical practice settings. The results of this study will expand on previous literature describing barriers to and facilitators of parent-integrated pain care [28] by specifically developing implementation interventions to support parental participation. Following the completion of this study, the identified implementation interventions will be pilot-tested and experimentally evaluated in subsequent research to understand their impact on parent integration in pain management in infants.

### Strengths

A strength of this study is that it is guided by implementation science theory to support the development of tailored implementation interventions. The TDF [36,37] and BCW [38] have been previously used to comprehensively map barriers to and facilitators of evidence use in health care [36,40] and develop implementation strategies to support evidence use [38]. The recruitment of a diverse sample of health care providers and parents will enhance the relevance of the findings. Inclusion of a parent partner and clinical stakeholders on the research team, as well as completion of advisory committee meetings to review and revise implementation interventions, will ensure that parent and clinician perspectives and voice are well represented. Given the strong theoretical foundation, the diverse sample, and purposive inclusion of stakeholder voice in this work, we anticipate that the developed implementation interventions will be successful in supporting parent-led pain care in infants in subsequent research.

### Limitations and Anticipated Challenges

The recruitment and retention of diverse and representative participants is a potential challenge that could impose limitations on this research. To proactively minimize this risk, we have specific support with participant recruitment through members of our research team (which includes health systems partners, a parent partner, and support from the Solutions for Kids in Pain, a Canadian knowledge mobilization network). We will provide all participants a gift card as an honorarium for taking part in the study interviews, and parent partners will be compensated for participation in the study advisory committee [56]. In addition, participants may experience additional or shifting workload demands and commitments as part of the response of the health and social system to the COVID-19 pandemic. As such, we have dedicated long time blocks in the work plan of our study to conduct study procedures and account for competing priorities.

### Conclusions

This protocol represents a theoretically informed and evidence-based approach to comprehensively understanding the barriers to and facilitators of parent-led pain care in infants and design implementation interventions to support best-practice pain care for infants. The successful integration of parents in pain care is crucial to support patient safety and positive health outcomes for diverse infants and families.

## Abbreviations

BCW: Behavior Change Wheel
NICU: neonatal intensive care unit
TDF: Theoretical Domains Framework

## References

[1] MacDonald TK (2019). Lactation care for transgender and non-binary patients: empowering clients and avoiding aversives. J Hum Lact.

[2] MacDonald T, Noel-Weiss J, West D, Walks M, Biener M, Kibbe A, Myler E (2016). Transmasculine individuals' experiences with lactation, chestfeeding, and gender identity: a qualitative study. BMC Pregnancy Childbirth.

[3] Van Wijlen JE, Aston M (2019). Applying feminist poststructuralism as a framework for exploring infant feeding interactions in the neonatal intensive care unit. Can J Crit Nurs Discourse.

[4] McMillan D, Canadian Paediatric Society, FetusNewborn Committee (1997). Routine administration of vitamin K to newborns. Paediatr Child Health.

[5] Stevenson DK, Fanaroff AA, Maisels MJ, Young BW, Wong RJ, Vreman HJ, MacMahon JR, Yeung CY, Seidman DS, Gale R, Oh W, Bhutani VK, Johnson LH, Kaplan M, Hammerman C, Nakamura H (2001). Prediction of hyperbilirubinemia in near-term and term infants. J Perinatol.

[6] Williams AF (2005). Neonatal hypoglycaemia: clinical and legal aspects. Semin Fetal Neonatal Med.

[7] Canadian immunization guide. Public Health Agency of Canada.

[8] Mörelius E, He H, Shorey S (2016). Salivary cortisol reactivity in preterm infants in neonatal intensive care: an integrative review. Int J Environ Res Public Health.

[9] Mörelius E, Theodorsson E, Nelson N (2005). Salivary cortisol and mood and pain profiles during skin-to-skin care for an unselected group of mothers and infants in neonatal intensive care. Pediatrics.

[10] Vinall J, Miller SP, Synnes AR, Grunau RE (2013). Parent behaviors moderate the relationship between neonatal pain and internalizing behaviors at 18 months corrected age in children born very prematurely. Pain.

[11] Taddio A, Shah V, Gilbert-MacLeod C, Katz J (2002). Conditioning and hyperalgesia in newborns exposed to repeated heel lances. JAMA.

[12] Gokulu G, Bilgen H, Ozdemir H, Sarioz A, Memisoglu A, Gucuyener K, Ozek E (2016). Comparative heel stick study showed that newborn infants who had undergone repeated painful procedures showed increased short-term pain responses. Acta Paediatr.

[13] Taddio A, Katz J, Ilersich AL, Koren G (1997). Effect of neonatal circumcision on pain response during subsequent routine vaccination. Lancet.

[14] Taddio A, Goldbach M, Ipp M, Stevens B, Koren G (1995). Effect of neonatal circumcision on pain responses during vaccination in boys. Lancet.

[15] Vinall J, Miller SP, Chau V, Brummelte S, Synnes AR, Grunau RE (2012). Neonatal pain in relation to postnatal growth in infants born very preterm. Pain.

[16] Doesburg SM, Chau CM, Cheung TP, Moiseev A, Ribary U, Herdman AT, Miller SP, Cepeda IL, Synnes A, Grunau RE (2013). Neonatal pain-related stress, functional cortical activity and visual-perceptual abilities in school-age children born at extremely low gestational age. Pain.

[17] Vinall J, Zwicker JG, Grunau RE, Chau V, Poskitt KJ, Brant R, Synnes AR, Miller SP (2015). ISDN2014_0158: early neonatal pain exposure and brain microstructure interact to predict neurodevelopmental outcomes at 18 months corrected age in children born very preterm. Int j dev neurosci.

[18] Ranger M, Synnes AR, Vinall J, Grunau RE (2014). Internalizing behaviours in school-age children born very preterm are predicted by neonatal pain and morphine exposure. Eur J Pain.

[19] Taddio A, Shah V, Atenafu E, Katz J (2009). Influence of repeated painful procedures and sucrose analgesia on the development of hyperalgesia in newborn infants. Pain.

[20] Larsson BA, Norman M, Bjerring P, Egekvist H, Lagercrantz H, Olsson GL (1996). Regional variations in skin perfusion and skin thickness may contribute to varying efficacy of topical, local anaesthetics in neonates. Paediatr Anaesth.

[21] Stevens B, Johnston C, Taddio A, Jack A, Narciso J, Stremler R, Koren G, Aranda J (1999). Management of pain from heel lance with lidocaine-prilocaine (EMLA) cream: is it safe and efficacious in preterm infants?. J Dev Behav Pediatr.

[22] Benoit B, Martin-Misener R, Latimer M, Campbell-Yeo M (2017). Breast-feeding analgesia in infants: an update on the current state of evidence. J Perinat Neonatal Nurs.

[23] Shah PS, Herbozo C, Aliwalas LL, Shah VS (2012). Breastfeeding or breast milk for procedural pain in neonates. Cochrane Database Syst Rev.

[24] Johnston C, Campbell-Yeo M, Disher T, Benoit B, Fernandes A, Streiner D, Inglis D, Zee R (2017). Skin-to-skin care for procedural pain in neonates. Cochrane Database Syst Rev.

[25] Benoit B, Campbell-Yeo M, Johnston C, Latimer M, Caddell K, Orr T (2016). Staff nurse utilization of kangaroo care as an intervention for procedural pain in preterm infants. Adv Neonatal Care.

[26] Orr T, Campbell-Yeo M, Benoit B, Hewitt B, Stinson J, McGrath P (2017). Smartphone and internet preferences of parents: information needs and desired involvement in infant care and pain management in the NICU. Adv Neonatal Care.

[27] Johnston C, Barrington KJ, Taddio A, Carbajal R, Filion F (2011). Pain in Canadian NICUs: have we improved over the past 12 years?. Clin J Pain.

[28] McNair C, Chinian N, Shah V, McAllister M, Franck LS, Stevens B, Burry L, Taddio A (2020). Metasynthesis of factors that influence parents' participation in pain management for their infants in the NICU. J Obstet Gynecol Neonatal Nurs.

[29] Palomaa A, Korhonen A, Pölkki T (2016). Factors influencing parental participation in neonatal pain alleviation. J Pediatr Nurs.

[30] Franck LS, Oulton K, Bruce E (2012). Parental involvement in neonatal pain management: an empirical and conceptual update. J Nurs Scholarsh.

[31] Marfurt-Russenberger K, Axelin A, Kesselring A, Franck LS, Cignacco E (2016). The experiences of professionals regarding involvement of parents in neonatal pain management. J Obstet Gynecol Neonatal Nurs.

[32] Taddio A, Shah V, Leung E, Wang J, Parikh C, Smart S, Hetherington R, Ipp M, Riddell RP, Sgro M, Jovicic A, Franck L (2013). Knowledge translation of the HELPinKIDS clinical practice guideline for managing childhood vaccination pain: usability and knowledge uptake of educational materials directed to new parents. BMC Pediatr.

[33] Richardson B, Falconer A, Shrestha J, Cassidy C, Campbell-Yeo M, Curran JA (2020). Parent-targeted education regarding infant pain management delivered during the perinatal period: a scoping review. J Perinat Neonatal Nurs.

[34] Taddio A, Shah V, Bucci L, MacDonald NE, Wong H, Stephens D (2018). Effectiveness of a hospital-based postnatal parent education intervention about pain management during infant vaccination: a randomized controlled trial. Can Med Assoc J.

[35] Wensing M, Bosch M, Grol R (2010). Developing and selecting interventions for translating knowledge to action. Can Med Assoc J.

[36] Atkins L, Francis J, Islam R, O'Connor D, Patey A, Ivers N, Foy R, Duncan EM, Colquhoun H, Grimshaw JM, Lawton R, Michie S (2017). A guide to using the Theoretical Domains Framework of behaviour change to investigate implementation problems. Implement Sci.

[37] Cane J, O'Connor D, Michie S (2012). Validation of the theoretical domains framework for use in behaviour change and implementation research. Implement Sci.

[38] Michie S, van Stralen MM, West R (2011). The behaviour change wheel: a new method for characterising and designing behaviour change interventions. Implement Sci.

[39] Cassidy C, Bishop A, Steenbeek A, Langille D, Martin-Misener R, Curran J (2018). Barriers and enablers to sexual health service use among university students: a qualitative descriptive study using the Theoretical Domains Framework and COM-B model. BMC Health Serv Res.

[40] Barker F, Atkins L, de Lusignan S (2016). Applying the COM-B behaviour model and behaviour change wheel to develop an intervention to improve hearing-aid use in adult auditory rehabilitation. Int J Audiol.

[41] Sandelowski M (2000). Whatever happened to qualitative description?. Res Nurs Health.

[42] Robinson OC (2013). Sampling in interview-based qualitative research: a theoretical and practical guide. Qual Res Psychol.

[43] Sandelowski M (1995). Sample size in qualitative research. Res Nurs Health.

[44] Guest G, Bunce A, Johnson L (2016). How many interviews are enough?. Field Method.

[45] Elo S, Kääriäinen M, Kanste O, Pölkki T, Utriainen K, Kyngäs H (2014). Qualitative content analysis. SAGE Open.

[46] Elo S, Kyngäs H (2008). The qualitative content analysis process. J Adv Nurs.

[47] Assarroudi A, Heshmati Nabavi F, Armat MR, Ebadi A, Vaismoradi M (2018). Directed qualitative content analysis: the description and elaboration of its underpinning methods and data analysis process. J Res Nurs.

[48] NVivo qualitative data analysis software. NVivo.

[49] Etherington C, Rodrigues IB, Giangregorio L, Graham ID, Hoens AM, Kasperavicius D, Kelly C, Moore JE, Ponzano M, Presseau J, Sibley KM, Straus S (2020). Applying an intersectionality lens to the theoretical domains framework: a tool for thinking about how intersecting social identities and structures of power influence behaviour. BMC Med Res Methodol.

[50] Shenton AK (2004). Strategies for ensuring trustworthiness in qualitative research projects. Educ Inform.

[51] Krefting L (1991). Rigor in qualitative research: the assessment of trustworthiness. Am J Occup Ther.

[52] Duncan E, O'Cathain A, Rousseau N, Croot L, Sworn K, Turner KM, Yardley L, Hoddinott P (2020). Guidance for reporting intervention development studies in health research (GUIDED): an evidence-based consensus study. BMJ Open.

[53] Buchanan F, Peasgood A, Easton M, Haas K, Narayanan U (2022). The Research Family Advisory Committee: the patient and family view of implementing a research-focused patient engagement strategy. Res Involv Engagem.

[54] Campbell-Yeo M, Dol J, Disher T, Benoit B, Chambers CT, Sheffield K, Boates T, Harrison D, Hewitt B, Jangaard K, Stinson J, Taddio A, Parker JA, Caddell K (2017). The power of a parent's touch: evaluation of reach and impact of a targeted evidence-based YouTube video. J Perinat Neonatal Nurs.

[55] Strategy for patient-oriented research patient engagement framework. Canadian Institutes of Health Research Strategy for Patient Oriented Research.

[56] Choosing methods for patient and caregiver engagement: a guide for health care organizations. Health Quality Ontario.

[57] Patient partner compensation policy. Solution for Kids in Pain.

